# Role of *Semisulcospira gottschei* extract as medicinal food on reflux esophagitis in rats

**DOI:** 10.1002/fsn3.2270

**Published:** 2021-05-06

**Authors:** Hyeon Hwa Nam, Sungyu Yang, Hyo Seon Kim, Min Jee Kim, Joong Sun Kim, Ji Hye Lee

**Affiliations:** ^1^ Herbal Medicine Resources Research Center Korea Institute of Oriental Medicine Naju Korea; ^2^ Present address: College of Korean Medicine Semyung University College of Korean Medicine Jecheon-si Chungcheongbuk-do

**Keywords:** inflammation, reflux esophagitis, Semisulcospira gottschei, snail

## Abstract

Gastroesophageal reflux disease (GERD) is a globally prevalent disease and results from a reflux of gastric contents into the esophagus. Existing synthetic drug‐based treatments for GERD have various drawbacks including refractory symptoms, relapse, or resistance due to long‐term use or may result in mucosal degeneration, polyps, and osteoporosis. *Semisulcospira gottschei* (SE), a freshwater snail, has been generally consumed as a food source due to its excellent flavor and nutritional value in Korea and considered to have therapeutic properties for various diseases including dyspepsia, stomachache, and hepatic diseases. The present study aims to investigate whether *Semisulcospira gottschei* extract (SGE) has a protective effect on reflux esophagitis‐induced rat models. The anti‐inflammatory effects of SGE were evaluated via NO production in LPS‐induced Raw 264.7 macrophage. And the protection effects of SGE were analyzed by assessing the amelioration of mucosal damage and expression of inflammation‐associated proteins in reflux esophagitis (RE) rats. Our results indicate that SGE significantly suppressed NO production in LPS‐induced raw 264.7 cells without any cytotoxicity. We observed mucosal lesions and histological changes in the esophagus of RE control rats. However, SGE treatment markedly ameliorated mucosal lesion ratio indicated through histological changes. SGE administration suppressed the expression of proteins related to inflammation, such as p‐NF‐κB, p‐IκBα, COX‐2, and TNF‐α, in esophageal tissue. Moreover, SGE elevated the expression of claudin‐5, which is a tight junction protein, involved in barrier function of epithelium and endothelium. The results suggest that SGE is useful as a medicinal food in esophagitis and may be helpful in developing effective treatment protocols for GERD.

## Graphical abstract

*Semisulcospira gottschei* extract demonstrates a protective effect on reflux esophagitis in gastroesophageal reflux disease (GERD), a globally prevalent disease. Treatment of reflux esophagitis rats markedly ameliorates mucosal lesion ratio indicated through histological changes, by suppressing inflammation‐related gene expression. Notably, *S. gottschei* extract can elevate the expression of tight junction protein, Claudin‐5, indicating an augmentation of the epithelium and endothelium to improve reflux esophagitis and present S. gottschei extract as a medicinal food source for the treatment of GERD.

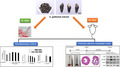

## 1 INTRODUCTION

Gastroesophageal reflux disease (GERD) is a prevalent disease of the gastrointestinal tract and is defined as a troublesome condition that results from a reflux of gastric contents into the esophagus (Vakil et al., 2006). The reflux of gastric contents is primarily caused by lower esophageal sphincter opening. Key factors that contribute to lower esophageal sphincter opening are lower esophageal sphincter relaxation, esophageal shortening, crural diaphragm inhibition, and a positive pressure gradient between the esophagogastric junction lumen and stomach (Pandolfino et al., 2006).

A typical symptom of GERD is heartburn caused due to acid regurgitation. GERD could also lead to several extraesophageal complications such as reflux esophagitis, laryngitis, and chronic cough (Vakil et al., 2006). Furthermore, persistent GERD is one of the risk factors for developing Barrett's esophagus and esophageal adenocarcinoma (Thrift, 2018).

The symptoms and complications of GERD also have a negative influence on the quality of life (Richter & Rubenstein, 2018). Several studies reported that patients with GERD showed lower health‐related quality of life score compared to healthy controls (Wood et al., 1998). As the worldwide prevalence of GERD has shown an increasing trend during the past decades (Mahant, 2017), it has caused an economic burden and health concern globally. The direct costs for the treatment of GERD were estimated to be approximately 9 to $10 billion per year in the USA (Shaheen et al., 2006).

The conventional agents generally used for treating GERD are proton pump inhibitors (PPIs) and H_2_‐receptor antagonists (Katz et al., 2013). Although these agents can rapidly relieve symptoms of GERD, they still have limitations associated with refractory symptoms, relapse, or resistance due to long‐term use (Eslami & Nasseri‐Moghaddam, 2013) (Subramanian & Triadafilopoulos, 2015). Especially, long‐term use of PPIs was related to development of mucosal degeneration, polyps, and osteoporosis (Chen et al., 2016), (Malfertheiner et al., 2017). Hence, there is a need to investigate new treatments for GERD with emphasis on safety, as well as clinical value.

*Semisulcospira gottschei* (SG) is a freshwater snail, widely distributed in Korea, China, Taiwan, and Japan (Davis, 1969). In Korea, SG has been generally consumed as a food source due to its excellent flavor and nutritional value (Shim et al., 1994). Besides nutritional aspect, SG has been considered to have therapeutic properties for various diseases including dyspepsia, stomachache, and hepatic diseases (Park et al., 2015). Recent studies have reported the bioactive effects of SG, such as antioxidant activity, anticancer activity, and hepatoprotective activity (Kim et al., 2009; Lee et al., 2010; Park et al., 2015). In this context, market demand for SG is increasing as a healthy dietary component (Moon et al., 2015). Until date, no studies have been carried out to assess the benefits of SG in GERD. The present study aims to investigate the protective effect of *Semisulcospira gottschei* extract (SGE) on reflux esophagitis (RE) using Raw 264.7 macrophage cells and surgically induced‐RE model in rats. The findings of our study will be helpful in highlighting the beneficial effects of this snail species as a natural resource for GERD treatment.

## 2 MATERIALS AND METHODS

### 2.1 Chemicals

Primary antibody of lamin B was purchased from Cell Signaling (Danvers, MA, USA), and all other antibodies and western luminal reagents were supplied by Santa Cruz. Bovine serum albumin protein kit was supplied by Bio‐Rad Laboratories. All other chemicals, of analytical reagent grade, were purchased from Sigma‐Aldrich.

### 2.2 Preparation of *Semisulcospira gottschei* extracts

SE was purchased from local fish market at Naju, Korea, and was morphologically authenticated by Dr. Goya Choi (Herbal Medicine Resources Research Center, Korea Institute of Oriental Medicine Naju, Korea). The voucher specimen (specimen number: 2‐19‐0398) was deposited at the Herbal Medicine Resources Research Center, Korea Institute of Oriental Medicine. Lyophilized and ground SE was extracted in distilled water for 3 hr under reflux (100 ± 2°C). After filtration of the extract solution, the extract was evaporated and then lyophilized using a freeze dryer to obtain the extract powder (41.52 g, yield 4.15%, w/w).

### 2.3 Cell viability in Raw 264.7 cells

Raw 264.7 cells (ATCC) were cultured in DMEM with 10% FBS and then incubated at 37°C under 5% CO_2_ in an incubator. The cells were transferred to 96‐well plates (1 × 10^6^ cells per well) and pretreated with SGE at concentrations of 0, 50, 1,000, and 2000 µg/ml. After 1 hr of pretreatment, the cells were treated with 1 µg/ml of LPS and incubated for 24 hr. Thereafter, cell viability was evaluated using a commercial cytotoxicity assay kit as per the manufacturer's instructions.

### 2.4 Nitric oxide production in Raw 264.7 cells

The amount of nitric oxide (NO) production was evaluated by the Griess reaction. The plate cultured with SGE and LPS‐induced Raw 264.7 cells was centrifuged for 5 min at 2,500 × *g*. The supernatant (50 µl) was mixed with 50 µl 0.1% NED and 1% sulfanilamide. After incubation at room temperature for 10 min, absorbance was measured at 540 nm. The amount of NO production was calculated using a standard curve of sodium nitrite.

### 2.5 Experimental animals and treatment

**FIGURE 1:**
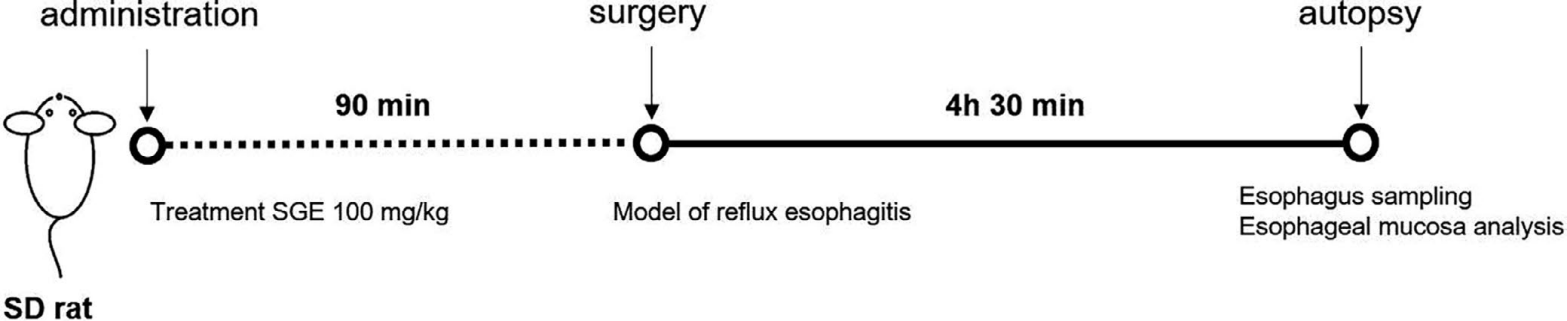
Summary of the experimental design

Seven‐week‐old male Sprague Dawley rats were supplied by Han‐il (Wanju, Korea). Rats were housed under 12‐hr light–dark cycle with controlled humidity (50 ± 5%), and temperature (23 ± 2°C), and ad libitum access to water and food. After adaptation for 1 week, the rats (*n* = 24) were randomly divided into four groups (*n* = 6 per group), viz. the normal, vehicle, SGE, and ranitidine groups, with no inter‐group differences in mean body weight. The normal and vehicle groups were given distilled water orally, whereas the SGE and ranitidine groups were orally administered with SGE (100 mg/kg) and ranitidine (40 mg/kg) for 90 min, respectively. All rats were fasted for 18 hr before surgery to induce reflux esophagitis; free access to water was provided. The surgery was performed following the method described previously (Choo & Roh, 2013), and all the subjects were sacrificed 4 hr 30 min after surgery (Figure 1). All experiments were conducted according to the “Guidelines for Animal Experimentation” approved by the Ethics Committee of Chonbuk National University (CBNU 2020‐010).

### 2.6 Esophageal lesion ratio

After sacrifice, the esophagus of each rat was longitudinally cut from the pharynx to the gastroesophageal junction. The dissected esophagus was photographed, and the images of esophageal lesions were analyzed by ImageJ program. Esophageal lesion ratio was calculated as follows:

$\text{Grosss esopahgeal mucosal damage ratio}\left(\%\right)=\frac{\text{area with esophageal mucosal damage}\;\left({\text{mm}}^{2}\right)}{\text{total area of esophagus}\;\left({\text{mm}}^{2}\right)}\times 100$

### 2.7 Histopathological analysis

For histopathological analyses, the esophageal tissue was immediately fixed in 10% neutral‐buffered formalin. After embedding in paraffin, the esophagus was sectioned into 5‐µm slices. The sectioned samples were stained using hematoxylin and eosin (H&E) and photographed using a Leica DM2500 microscope.

### 2.8 Western blot analysis

Cytosolic and nuclear fractions of the esophageal cells were conducted as described previously (Nam et al., 2018). The nuclear and cytosol fractions were electrophoresed by 12% and 10% sodium dodecylsulphate–polyacrylamide gel electrophoresis, respectively, and transferred to nitrocellulose membranes. After 1 hr blocking in 5% skimmed milk at room temperature, the membranes were incubated overnight at 4°C with the primary antibodies (1:1,000) of COX‐2, iNOS, claudin‐5, TNF‐α, lamin B, phospho‐NF‐κB, phospho‐IκB‐α, and β‐actin, respectively. Thereafter, the membranes were incubated with secondary antibodies (1:10,000) for 2 hr. Blots were visualized by Western blotting luminal reagent and detected using a ChemiDoc^TM^ MP imaging system (Bio‐Rad).

### 2.9 Statistical analysis

Data were expressed as mean ± SD. Statistical analysis was conducted by one‐way analysis of variance (ANOVA) followed by Tukey's multiple comparison tests using SPSS software (SPSS Inc). Differences were considered significant at *p* < .05.

## 3 RESULTS AND DISCUSSION

### 3.1 Raw 264.7 cell viability

**FIGURE 2:**
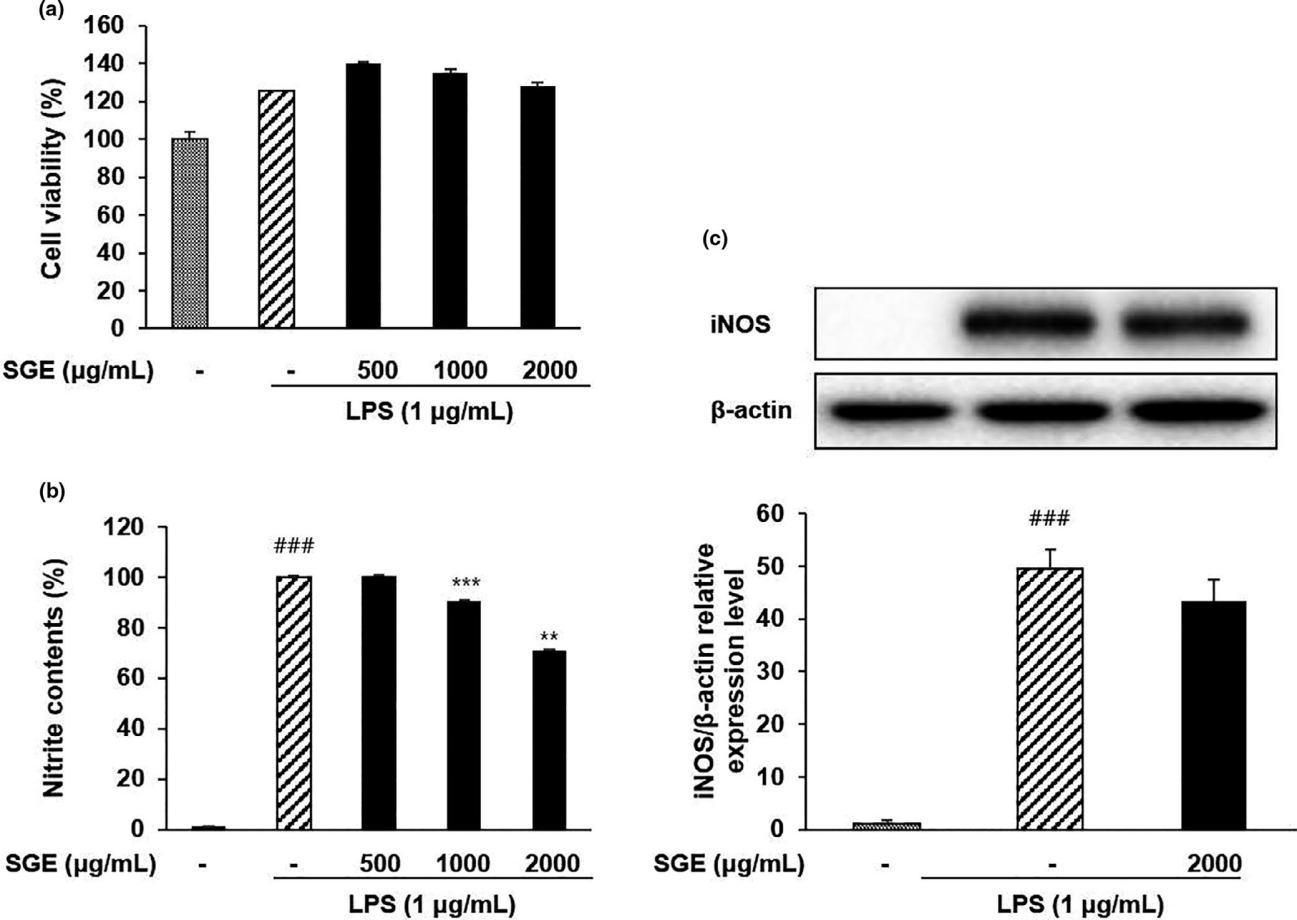
Effect of SGE on cell viability (a), NO production (b), and iNOS expression (c) in Raw 264.7 cells. Raw 264.7 cells were treated with SGE (500, 1,000, 2000 μg/ml) and LPS (1 μg/ml) for 18 hr. ^###^ *p* <.001 versus normal cells; ^***^ *p* < .001 and ^**^ *p* < .01 versus LPS‐induced cells

The cytotoxicity of SGE in Raw 264.7 cells treated with LPS has been presented in Figure 2a. The results of cytotoxicity assay indicated that the SGE, up to the concentration of 2000 μg/ml, causes no cytotoxicity to the Raw 264.7 cells.

### 3.2 NO production and iNOS expression in LPS‐induced Raw 264.7 cells

LPS, a major component in outer membrane of Gram‐negative bacteria, is generally used to induce inflammation for in vivo and in vitro models (Bhardwaj et al., 2020), (Kim et al., 2020; Deng et al., 2021). LPS activates macrophages and involves the expression of pro‐inflammatory cytokines and mediators including iNOS (Nicholas et al., 2007). The overexpressed iNOS generates excessive NO, which plays an important role in inflammation and regulation of immune responses (Speyer et al., 2003) (Guzik et al., 2003). Excessive NO could also cause tissue dysfunction and DNA damage (Speyer et al., 2003) (McAdam et al., 2012). Therefore, we used LPS‐induced Raw 264.7 cells to evaluate the anti‐inflammatory activity of SGE. As shown in Figure 2b and c, NO production is increased by the treatment with LPS 1 μg/ml compared to untreated cells. However, the SGE treatment significantly suppressed the level of NO production in a concentration‐dependent manner in the range of 500–2000 μg/ml. The expression of iNOS protein was decreased by SGE, but there is no significant difference compared with LPS‐induced cells without SGE treatment.

### 3.3 Esophageal lesion ratio

**FIGURE 3:**
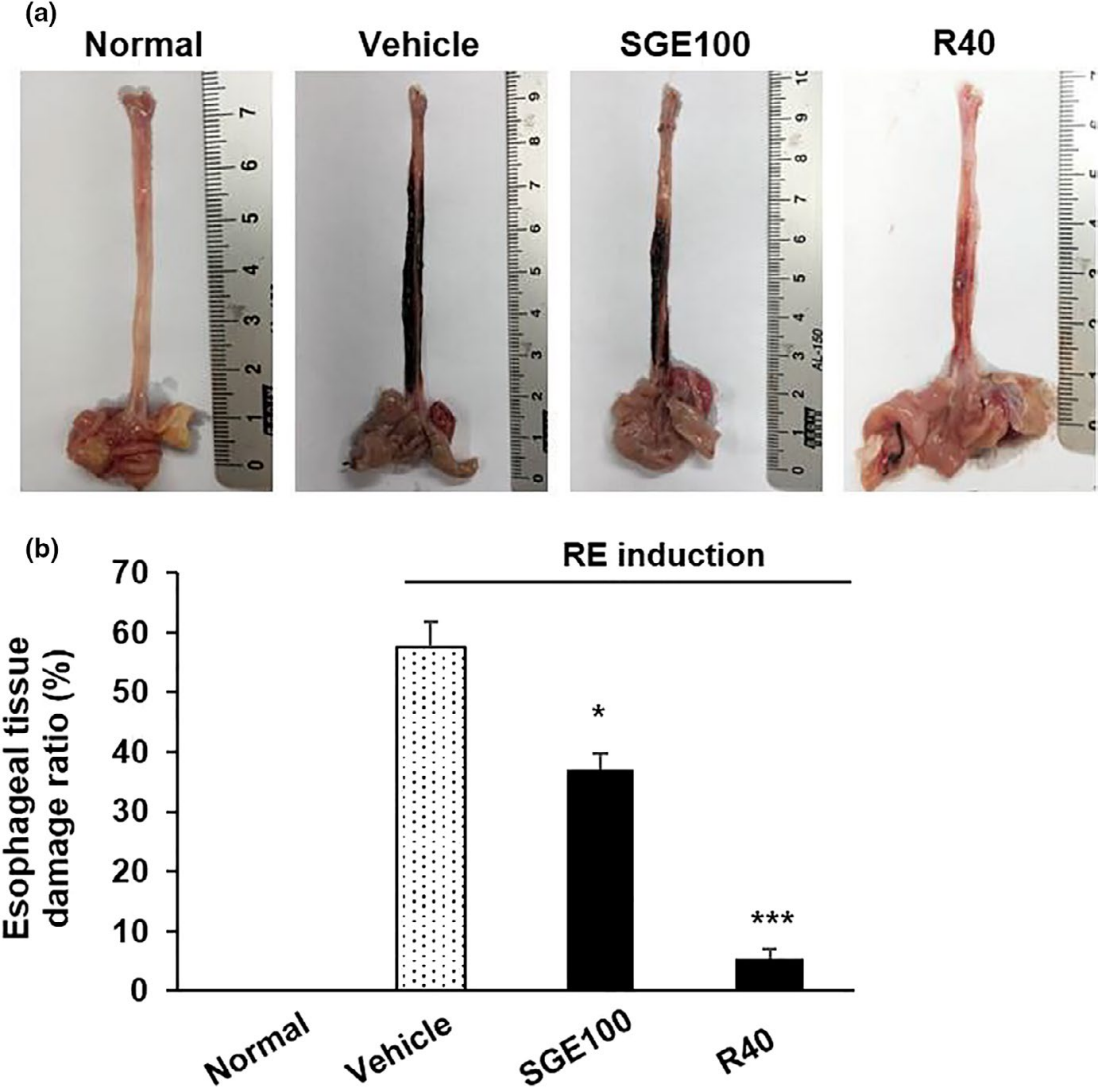
Effects of SGE on esophagus tissues of rats with induced esophagitis. The images of esophagus mucosa (a) and esophageal tissue damage ratio (b). Normal: Normal group; Vehicle: induced esophagitis group; SGE100: RE +SGE 100 mg/kg group; R40: RE +ranitidine 40 mg/kg. ^***^ *p* < .001 and ^*^ *p* < .05 versus vehicle group

The protective effects of SGE on reflux esophagitis were demonstrated using the experimental RE rat model. In the control group, several damages such as mucosal lesions, loss of esophagus pits, and hemorrhage were present on the mucosal surface of the esophagus, while the normal rats had any detectable damage on esophageal mucosa. Meanwhile, SGE administration markedly preserved esophageal mucosa compared to RE control rats (Figure 3a and b).

### 3.4 Histopathological changes in the esophagus

**FIGURE 4:**
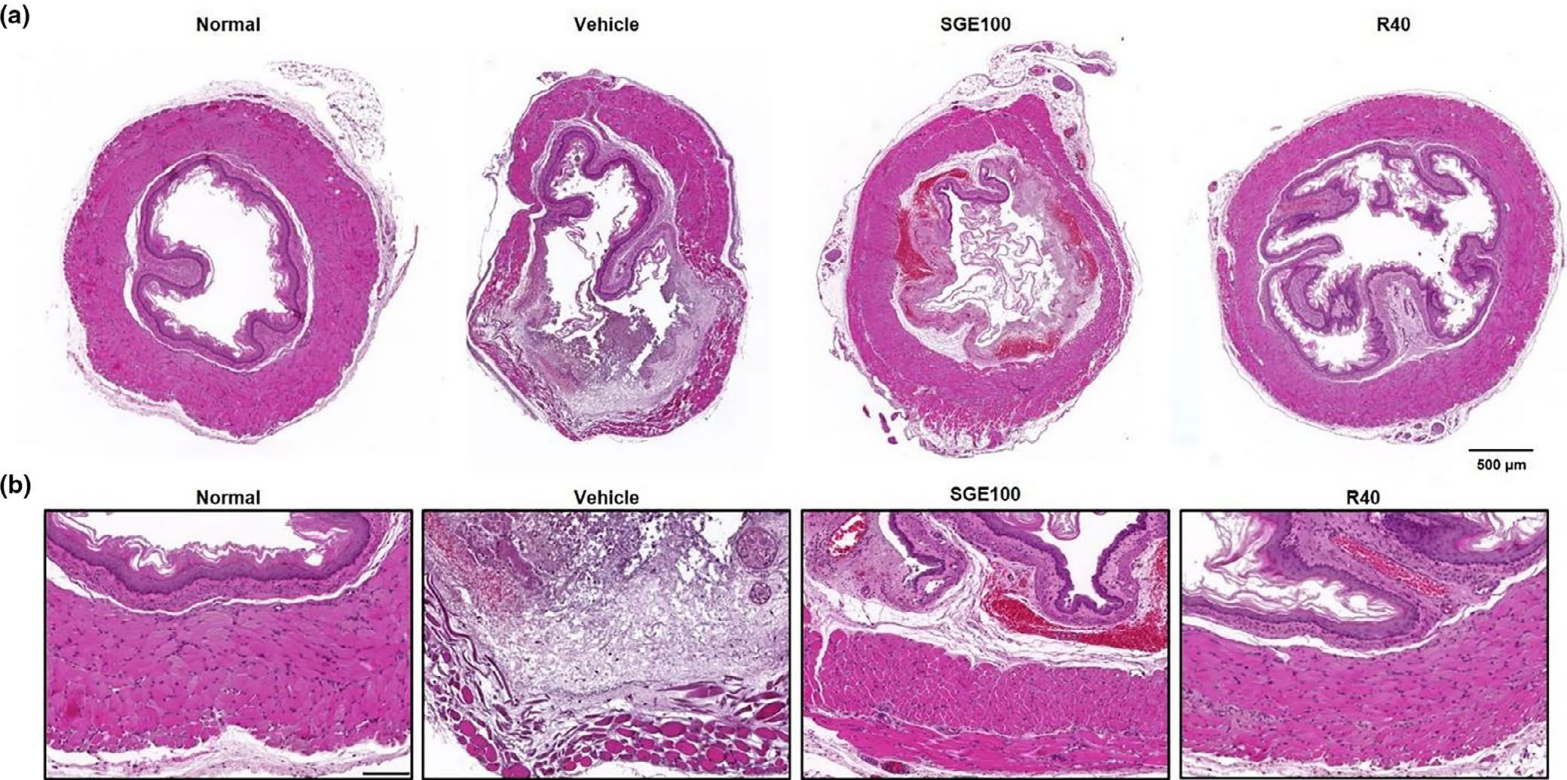
Histopathological changes of esophageal mucosa in reflux esophagitis rats by hematoxylin and eosin staining. The histological change evaluation in different group: Normal: Normal group; Vehicle: induced esophagitis group; SGE100: RE +SGE 100 mg/kg group; R40: RE +ranitidine 40 mg/kg

Histological changes in mucosa due to esophagitis have been presented in Figure 4. The histological changes such as esophageal ulcer area and mucosa inflammation may be caused by reflux of the gastric contents and acid (De Araújo et al., 2018). After the surgery, we could observe histological changes such as the thin epithelial layer, inflammatory cell infiltration, and thin epithelial layer in RE control rats. However, the administration of SGE effectively improved the esophageal damages ratio and histological changes. In the present result, pretreatment with SGE was able to protect the mucosa in esophagus lesion induction model.

### 3.5 Tight junction protein expression in the esophagus

**FIGURE 5:**
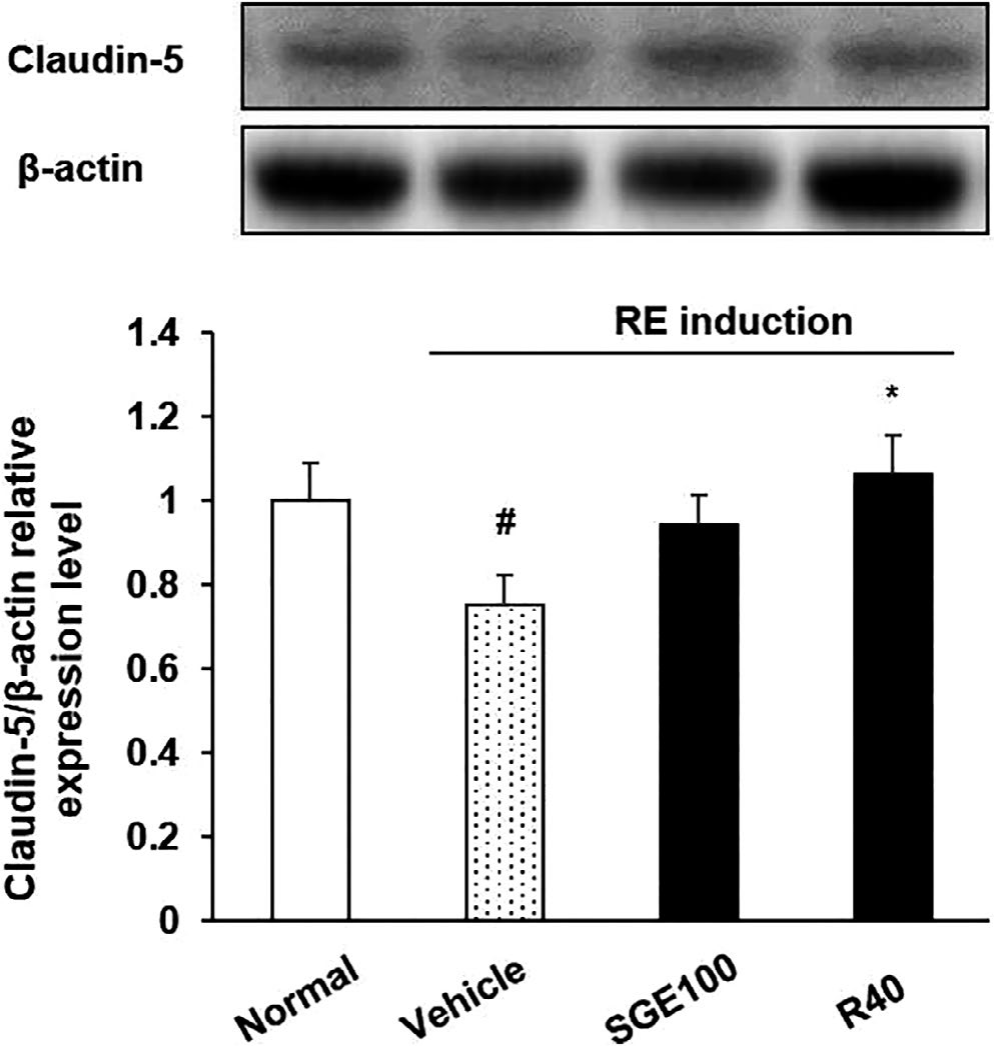
The effect of SGE on claudin‐5 protein expression level in reflux esophagitis (RE) rats. Normal: Normal group; Vehicle: induced esophagitis group; SGE100: RE +SGE 100 mg/kg group; R40: RE +ranitidine 40 mg/kg. ^#^ *p* < .05 versus normal group; ^*^ *p* < .05 versus vehicle group

The pathogenesis of GERD is associated with increased paracellular permeability of esophageal epithelium induced by gastric acid (Jovov et al., 2011) (Björkman et al., 2012). Claudin, an integral membrane tight junction protein, plays a major role in barrier function in the epithelium and endothelium. Previous studies reported that the expression of claudin increased when the lesions occurred in esophageal mucosa (Gweon et al., 2018). As shown in Figure 5, the level of claudin‐5 expression significantly decreased in RE control rats, which presented the severe damage in esophageal tissue. However, the SGE administration significantly induced a higher level of claudin‐5 in the esophagus.

### 3.6 Expression of p‐IκBα and p‐NF‐κB in the esophagus

**FIGURE 6:**
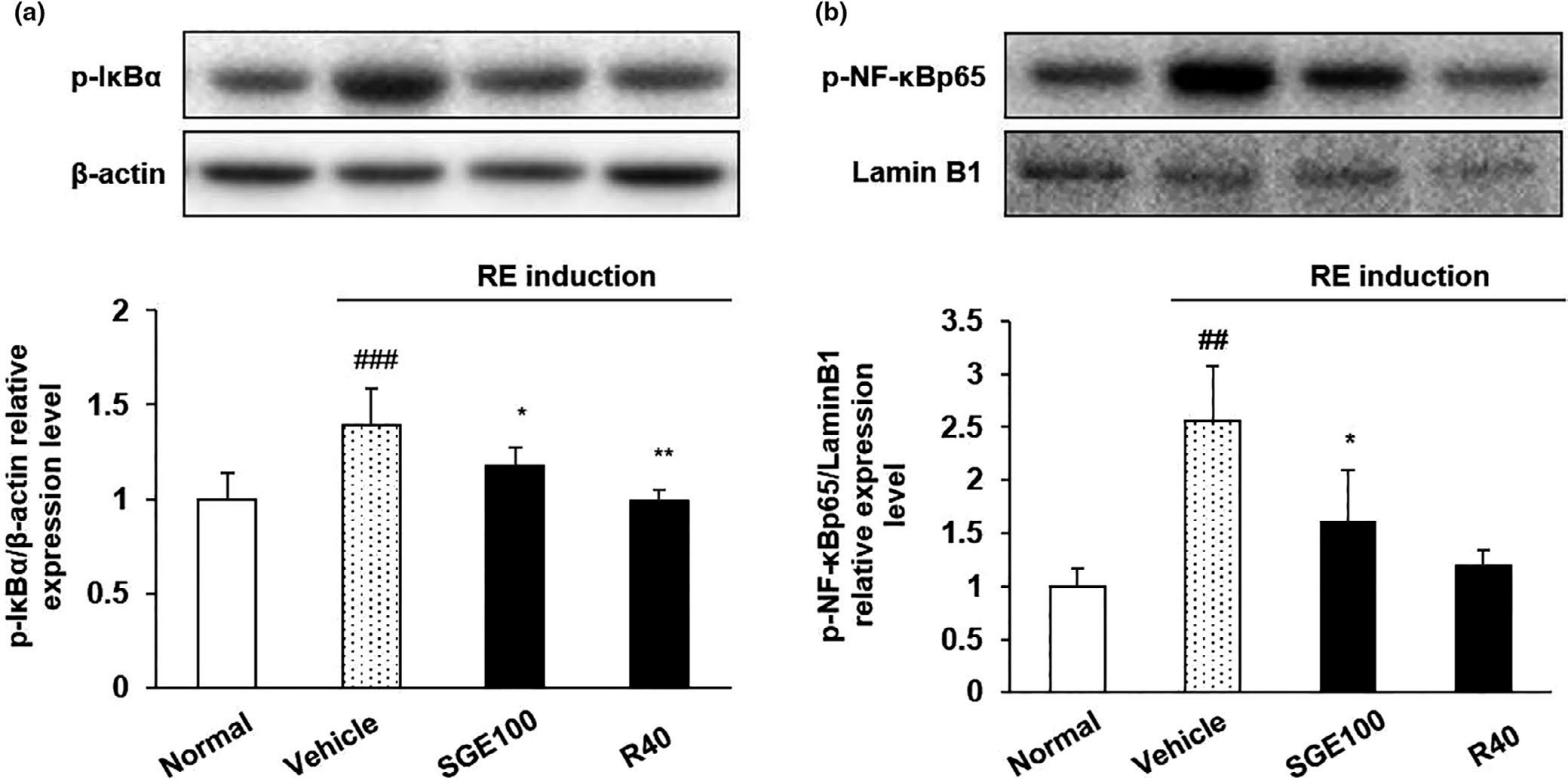
Inhibitory effects of SGE on the expression of p‐IκBα and p‐NF‐κBp65 in esophagus tissues. Normal: Normal group; Vehicle: induced esophagitis group; SGE100: RE +SGE 100 mg/kg group; R40: RE +ranitidine 40 mg/kg. ^###^ *p* < .001 versus normal group; ^##^ *p* < .01 versus normal group; ^**^ *p* < .01 and ^*^ *p* < .05 versus vehicle group

Several studies have reported that the expression of proteins related to inflammation is reduced in the esophagus when esophageal mucosa damage is ameliorated (Souza et al., 2009). NF‐κB is one of transcriptional factors involved in the expression of pro‐inflammatory mediator and cytokines during inflammatory responses (Ghosh & Karin, 2002). NF‐κB is sequestered in cytoplasm by binding with IκBα, an inhibitor protein. Under inflammatory responses, IκBα is phosphorylated and degraded, leading to the release of NF‐κB, which gets translocated to the nucleus and binds to the promoter regions of target genes (Rothwarf & Karin, 1999). Thus, targeting NF‐κB has been focused upon to develop drugs for inflammatory diseases, including GERD (Karin et al., 2004). The levels of inflammation‐related proteins p‐IκBα and p‐NF‐κB regulate the exposure to pro‐inflammatory proteins and cytokines (Salka et al., 2020). The level of p‐NF‐κB and p‐IκBα expression in RE control rats was significantly higher than untreated rats (Figure 6a and b). Conversely, the expression of these proteins was significantly lowered by SGE administration.

### 3.7 Expression of COX‐2 and TNF‐α in the esophagus

**FIGURE 7:**
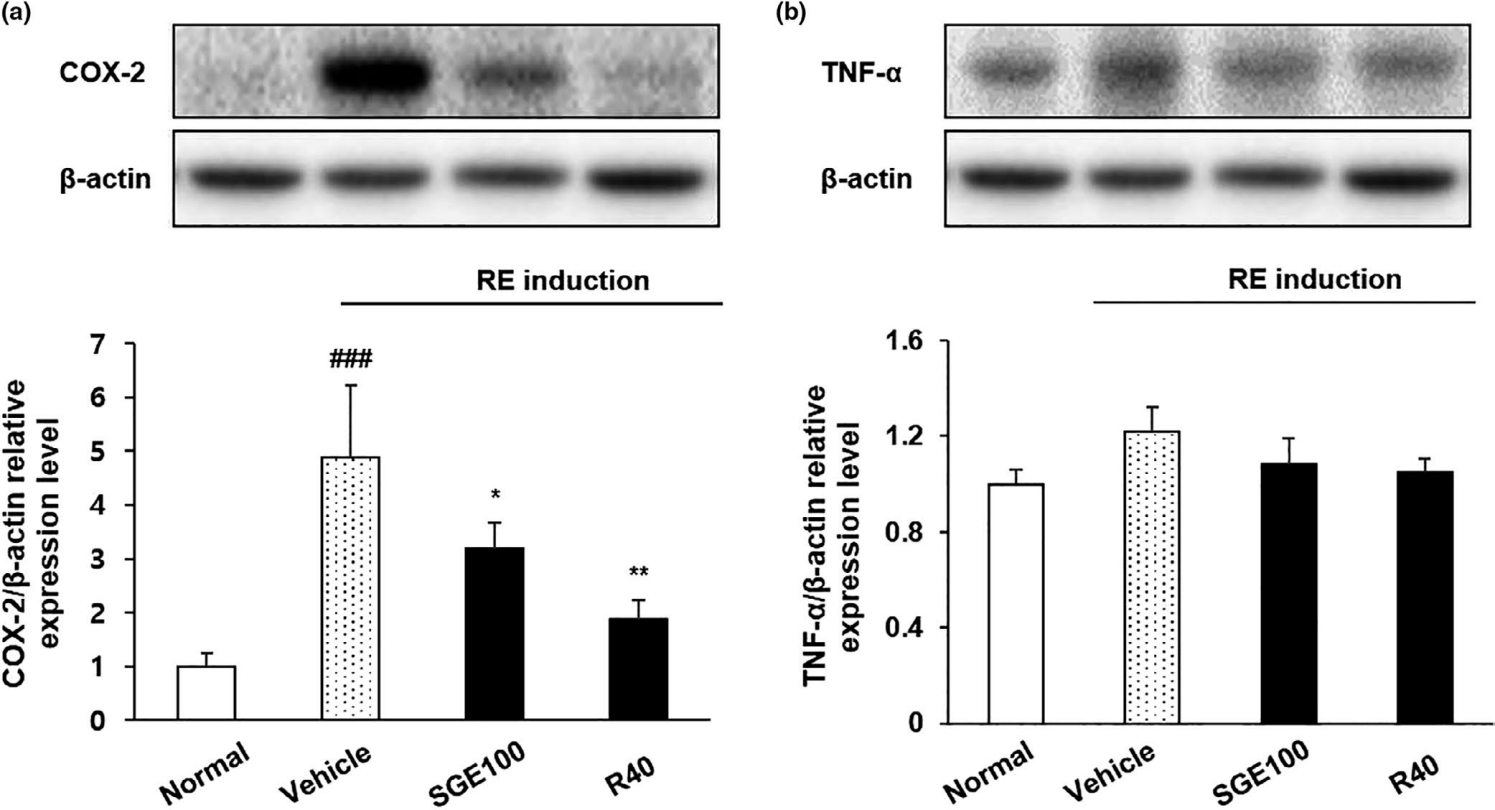
Inhibitory effects of SGE on the expression of COX‐2 protein (a) and TNF‐α cytokine (b) in the esophagus. Normal: Normal group; Vehicle: induced esophagitis group; SGE100: RE +SGE 100 mg/kg group; R40: RE +ranitidine 40 mg/kg. ^###^ *p* < .001 versus normal group; ^**^ *p* < .01 and ^*^ *p* < .05 versus vehicle group

Activated NF‐κB regulates various pro‐inflammatory mediators including COX‐2 (Rothwarf & Karin, 1999). Furthermore, under inflammatory conditions, macrophages, and monocyte secrete cytokines including TNF‐α, which induces pleiotropic physiological effects such as septic shock, inflammation, and cytotoxicity (Cao et al., 2004) (Arango Duque & Descoteaux, 2014). To investigate the anti‐inflammatory effect of SGE on esophageal tissues, the levels of COX‐2 and TNF‐α were measured (Figure 7a and b). We observed the increased level of COX‐2 expression in RE control rats, while SGE administration significantly suppressed excessive COX‐2 expression. Previous studies reported that the expression of COX‐2 was increased in esophageal tissue of RE rat model (Buttar et al., 2002). Thus, in the present study, reflux esophagitis increased the level of COX‐2 expression, along with TNF‐α expression. On the contrary, the SGE administration significantly suppressed the COX‐2 expression. The increased TNF‐α in the RE rats was slightly decreased by SGE administration, but there was no significant change compared with RE control rats. Therefore, the result of our study showed that SGE protects the esophageal mucosa in esophagitis induced rats by regulating the activation of TNF‐α and COX‐2, along with p‐NF‐κB and p‐IκBα discussed earlier.

## 4 CONCLUSION

Due to the worldwide prevalence of GERD showing an increasing trend during the past decades, GERD has emerged as a major health concern (Mahant, 2017). Furthermore, GERD also causes an economic burden; the cost of treating diseases in patients with GERD has been assumed to be twofold more costly than comparable individuals without it (Bloom et al., 2001). Despite advances in medicine, the limitations in treating RE by means of drugs still persist due to undesirable adverse effects, relapse, and resistance to long‐term use (Eslami & Nasseri‐Moghaddam, 2013) (Subramanian & Triadafilopoulos, 2015). As traditional medicine is highlighted as a potential source for new drugs, several studies have investigated the therapeutic effects of traditional medicine on RE, and several natural resource have been reported to ameliorate the effects of gastrointestinal diseases (Nam et al., 2018), (Shin et al., 2017), (Kim et al., 2020), (Kengkoom et al., 2017), (Miao et al., 2021), (Yasin et al., 2020; Li et al., 2020). The present study attempted to investigate whether SG, a functional food in Korea, has any protective effect on RE, using LPS‐induced Raw 264.7 cells and RE induced rat models. Our results indicate that the administration of SGE could ameliorate esophageal damage. The speculated mechanism seems to inhibit the NF‐κB inflammation pathway and improve sealing functions in esophageal tissue. These results imply that SGE possesses the potential to be a new agent or supplement for treating GERD. To substantiate the efficacy of SGE on RE, further studies are required, which investigate the activity depending on dose and the detailed mechanism of action.

## CONFLICT OF INTEREST

The authors declare that they do not have any conflict of interest.

## ETHICAL APPROVAL

All experiments were conducted according to the “Guidelines for Animal Experimentation” approved by the Ethics Committee of Chonbuk National University (CBNU 2020‐010).

## INFORMED CONSENT

Written informed consent was obtained from all study participants.
